# BRCA1-deficient mammary tumor cells are dependent on EZH2 expression and sensitive to Polycomb Repressive Complex 2-inhibitor 3-deazaneplanocin A

**DOI:** 10.1186/bcr2354

**Published:** 2009-08-26

**Authors:** Julian Puppe, Rinske Drost, Xiaoling Liu, Simon A Joosse, Bastiaan Evers, Paulien Cornelissen-Steijger, Petra Nederlof, Qiang Yu, Jos Jonkers, Maarten van Lohuizen, Alexandra M Pietersen

**Affiliations:** 1 Molecular Genetics and Cancer Genomics Centre, Netherlands Cancer Institute, Plesmanlaan 121, 1066 CX, Amsterdam, The Netherlands; 2 Institute of Physiological Chemistry, The Medical Faculty Carl-Gustav Carus, Fetscherstraß 74, 01307 Dresden, Germany; 3 Division of Molecular Biology, Netherlands Cancer Institute, Plesmanlaan 121, 1066 CX, Amsterdam, The Netherlands; 4 Department of Experimental Therapy, Netherlands Cancer Institute, Plesmanlaan 121, 1066 CX, Amsterdam, The Netherlands; 5 Department of Pathology, Netherlands Cancer Institute, Plesmanlaan 121, 1066 CX, Amsterdam, The Netherlands; 6 Molecular Pharmacology, Genome Institute of Singapore, 60 Biopolis Street, 138672 Singapore; 7 National Cancer Centre Singapore/Duke-NUS GMS, 11 Hospital Drive, 169610 Singapore; 8 Current address: Biomedical Analysis Center, Tsinghua University, Beijing 100084, PR China

## Abstract

**Introduction:**

Treatment of breast cancer is becoming more individualized with the recognition of tumor subgroups that respond differently to available therapies. Breast cancer 1 gene (BRCA1)-deficient tumors are usually of the basal subtype and associated with poor survival rates, highlighting the need for more effective therapy.

**Methods:**

We investigated a mouse model that closely mimics breast cancer arising in *BRCA1*-mutation carriers to better understand the molecular mechanism of tumor progression and tested whether targeting of the Polycomb-group protein EZH2 would be a putative therapy for BRCA1-deficient tumors.

**Results:**

Gene expression analysis demonstrated that EZH2 is overexpressed in BRCA1-deficient mouse mammary tumors. By immunohistochemistry we show that an increase in EZH2 protein levels is also evident in tumors from *BRCA1*-mutation carriers. EZH2 is responsible for repression of genes driving differentiation and could thus be involved in the undifferentiated phenotype of these tumors. Importantly, we show that BRCA1-deficient cancer cells are selectively dependent on their elevated EZH2 levels. In addition, a chemical inhibitor of EZH2, 3-deazaneplanocin A (DZNep), is about 20-fold more effective in killing BRCA1-deficient cells compared to BRCA1-proficient mammary tumor cells.

**Conclusions:**

We demonstrate by specific knock-down experiments that EZH2 overexpression is functionally relevant in BRCA1-deficient breast cancer cells. The effectiveness of a small molecule inhibitor indicates that EZH2 is a druggable target. The overexpression of EZH2 in all basal-like breast cancers warrants further investigation of the potential for targeting the genetic make-up of this particular breast cancer type.

## Introduction

Breast cancer is a heterogeneous disease. Studies by Perou and colleagues and Sorlie and colleagues have demonstrated that at least five different subtypes can be identified based on molecular profiling [1,2]. These different subtypes might arise from transformation of different cell types in the breast and/or from mutations in different genes. It has become clear that breast cancer subtypes correspond with marked differences in therapy response and overall survival, indicating that each subgroup should be treated differently [3]. To a certain extent this is already common practice, as ErbB2-overexpressing tumors are treated with herceptin and estrogen receptor (ER)-positive tumors with tamoxifen or aromatase inhibitors [4]. However, for other groups, such as the basal-type tumors that lack expression of ErbB2, ER, and progesterone receptor (PR), rationally designed treatments are currently lacking. These tumors are generally characterized by a poor differentiation grade, and it is speculated that they may arise from an undifferentiated breast epithelial cell, or at least have acquired stem cell-like properties during transformation [5]. Currently, standard treatment of these tumors is chemotherapy. Although there is an initial effect of chemotherapy agents such as anthracyclins, basal-like tumors nevertheless exhibit the worst overall survival rate of all breast cancer subtypes. This highlights the need for more effective therapies.

In the current study, we investigated the potential of a molecular-based therapy for a subgroup of basal-like breast tumors: those arising in women with an inherited mutation in *BRCA1*. These tumors are characterized by the loss of the second *BRCA1 *allele, concomitant loss of *TP53 *function and an undifferentiated, basal-like phenotype [6-9]. Consistent with their basal-like characteristics, BRCA1-deficient breast tumors exhibit aggressive behavior and are associated with poor survival. At the cellular level, an important consequence of loss of BRCA1 function is impaired DNA double-strand break repair [10]. As unresolved double-strand breaks will activate p53, resulting in either cell cycle arrest or apoptosis, there is a strong selection pressure on loss of p53 function in BRCA1-associated breast tumorigenesis. In addition, recent evidence indicates that loss of BRCA1 inhibits differentiation into ER-positive luminal cells, which might contribute to the undifferentiated phenotype [11].

We developed a mouse model mimicking human BRCA1-deficient breast cancer to gain insight into the molecular progression of BRCA1-deficient tumors and to test putative therapies [12]. In this model, the *Brca1 *and *p53 *genes are deleted by tissue-specific expression of Cre recombinase driven by the keratin 14 promoter, which is active in basal cells of the mammary gland, including the stem cells [13]. The ensuing mammary tumors show a solid growth pattern with pushing margins, and are highly proliferative, poorly differentiated and similar to human basal-like breast cancers (ER-, PR- and human epidermal growth factor receptor (HER) 2-negative).

Importantly, our mouse model allows us to compare BRCA1-deficient mammary tumors (arising in *K14cre;Brca1*^*F*/*F*^*;p53*^*F*/*F *^(KB1P) mice) with BRCA1-proficient control tumors (arising in *K14cre;Brca1*^*w*.*t*/*w*.*t*^*;p53*^*F*/*F *^(KP) mice). After comparing gene expression patterns of BRCA1-deficient mouse mammary tumors with BRCA1-proficient control tumors, we noted that *Ezh2 *expression was particularly high in BRCA1-deficient tumors. EZH2 is a member of the family of polycomb group proteins, which are epigenetic repressors that prevent the expression of cell cycle inhibitors and genes required for differentiation [14,15]. We and others have already observed that *EZH2 *overexpression is linked to aggressive tumours with a high proliferation rate and a poor prognosis [16-20].

In the study presented here, we set out to determine whether increased EZH2 expression also characterizes human BRCA1-deficient breast cancer, and whether BRCA1-deficient tumor cells are dependent on high EZH2 levels for their survival. This would indicate that EZH2 constitutes a therapeutic target for BRCA1-deficient breast cancer. EZH2 is the catalytic subunit of Polycomb Repressive Complex 2 (PRC2), which also contains SUZ12 and EED, and initiates gene silencing by trimethylating lysine 27 in histone H3 (H3-K27me3) [21]. Tan and colleagues recently demonstrated that a small molecule inhibitor, 3-deanzaneplanocin A (DZNep), effectively reduced the protein levels of PRC2 components EZH2, SUZ12, and EED, and inhibits the associated H3K27 trimethylation activity [22]. H3-K27me3 depletion resulted in reactivation of PRC2-silenced genes and apoptotic cell death in several cancer cell lines. The availability of a small molecule inhibitor such as DZNep allowed us to test whether pharmacological targeting of EZH2 function provides a selective approach to kill BRCA1-deficient breast tumor cells.

## Materials and methods

### Derivation and maintenance of mouse tumor cell lines

Tumor cell lines were generated from individual tumors arising in female mice with a KB1P or KP genotype as described previously [23]. Established cell lines were cultured at 37°C with 5% carbondioxide in DMEM-F12 medium (Gibco, Carlsbad, CA, USA) supplemented with 10% FCS, 50 U/ml penicillin, 50 μg/ml streptomycin (Gibco, Carlsbad, CA, USA), 5 μg/ml insulin (Sigma, St. Louis, MO, USA), 5 ng/ml epidermal growth factor (Invitrogen, Carlsbad, CA, USA) and 5 ng/ml cholera toxin (Gentaur, Brussels, Belgium).

### BRCA1 reconstitution in BRCA1-deficient tumor cells

One million KB1P3.12 cells were electroporated (3 μF, 0.8 kV) with the bacterial artificial chromosome (BAC) clone RP11-812O5 containing the complete human *BRCA1 *gene and regulatory sequences. The RP11-812O5 BAC was obtained from the Children's Hospital and Research Center at Oakland, CA, USA [24]. The vector backbone was modified by insertion of the pgk/EM7 Neo/KanR-positive selection cassette pCEI1 [25] into the sacBII gene by bacterial homologous recombination in *Escherichia coli *SW102 [26]. After selection with 300 μg/ml Geneticin (Gibco-BRL, Carlsbad, CA, USA) for two weeks, clones were picked and checked for the presence of the BAC by PCR for exon 11 of human BRCA1 [27].

### Tumorsphere formation assay

Stem cell medium (SCM) containing defined growth factors as described by [28,29] was freshly prepared each time and DZNep (5 μm) or dimethyl sulfoxide (DMSO; no-drug control) was added. Cells were trypsinized, which was inactivated with 10% serum and subsequently washed with PBS to remove the serum, and resuspended in SCM. Cells were filtered to obtain single cells and 40,000 cells, counted with a Casy counter (Schaerfe Systems, Reutlingen, Germany), were plated out in ultra-low binding plates with a flat bottom (Corning Incorporated, Corning, NY, USA). Sphere formation was checked every day and cell culture images were obtained after 72 hours using a Zeiss Axiovert 25 microscope (Carl Zeiss MicroImaging, Goettingen, Germany) with 10× objective on a Sony Cybershot (Sony corporation, Tokyo, Japan).

### Classification of human breast tumor samples

Human breast tumor tissue samples were obtained from the pathology archive of the Netherlands Cancer Institute. *BRCA1*-mutation status was determined by routine DNA diagnostics. The basal-like and luminal status was determined using expression data to classify the tumors according to the intrinsic gene set as described [30].

### Immunohistochemistry

Paraffin-embedded tumor samples were sectioned (4 μm) and deparaffinized by treating twice with xylene for 10 minutes each and subsequently hydrated in 100%, 80%, and 70% ethanol. Antigen retrieval was performed by boiling samples in 10 mM sodium citrate for one minute at 900 W and 15 minutes at 250 W in the microwave, followed by 20 minutes gradual cooling at room temperature. Slides were blocked in 5% normal goat serum in PBS. Mouse tissue was additionally permeabilized with 0.25% Triton prior to blocking. The samples were incubated overnight with a mouse monoclonal antibody against EZH2 (1:100, BD Biosciences San Jose, California, USA). Immunodetection was performed by diaminobenzidine (DAB) oxidation using the Powervision system (ImmunoLogic, Duiven, The Netherlands). Samples were dehydrated in 70%, 80%, and 100% ethanol. Once immunostained, slides were digitally scanned with a Scanscope (Aperio Technologies, Vista, CA, USA) and the amount of EZH2-expressing epithelial cell nuclei were counted in a blinded manner by two observers (SAJ and JP) independently. For statistical analysis, the Wilcoxon test was used to compare the percentage of EZH2-positive nuclei between two samples and *P *< 0.05 was considered statistically significant.

### Immunoblot analysis

Cells were scraped from subconfluent plates and lysed in RIPA buffer (150 mM NaCl, 10 mM Tris-HCl pH 7.5, 1% Triton X100, 1% DOC, 0.1% SDS, 1 mM EDTA) Equal amount of protein (10 μg) was loaded and separated by electrophoresis on NuPAGE Novex 4 to 12% SDS-PAGE (Invitrogen, Carlsbad, CA, USA) and transferred to nitrocellulose membranes. The blot was blocked with TBST (0.1% Tween 20) containing 5% BSA (Sigma-Aldrich, St. Louis, MO, USA) and incubated with primary antibodies for two hours at room temperature. After washing with TBST, the membrane was incubated with a horseradish peroxidase (HRP)-conjugated secondary antibody and the signal was detected with enhanced chemiluminescence substrate (GE Healthcare, Amersham, UK). Following antibodies were used: mouse monoclonal against EZH2 (1:20, provided by K. Helin), β-tubulin (1:10000, Sigma-Aldrich, St. Louis, MO, USA), anti-mouse IgG-HRP (1:10000, Biosource International, Camrillo, CA, USA).

### Quantitative real-time PCR

Total RNA was extracted using Trizol Reagent (Invitrogen, Carlsbad, CA, USA) and 1 μg per sample was treated with DNase. Reverse transcription was performed using the SuperScript™ First-Strand Synthesis System for RT-PCR (Invitrogen, Carlsbad, CA, USA; according to manufacturer's protocol). The generated cDNA was analyzed using SYBR Green (Taqman universal PCR master mix, Applied Biosystems, Foster City, CA, USA), performed on an ABI Prism 7000 SDS (Applied Biosystems, Foster City, CA, USA). Product accumulation was evaluated using the comparative C_T _method (DDC_T_), with Hprt levels as internal control for normalization. The following primers were used (all for mouse transcripts): *Hprt *FW: CTGGTGAAAAGGACCTCTCG; *Hprt *RV: TGAAGTACTCATTATAGTCAAGGGCA, *Ezh2 *FW: AAAGACCCTGAATGCAGTCGC; *Ezh2 *RV: TGATCCAGAACTTCATCCCCC.

### Microarray analysis

Expression values (Log2 ratio) of *Ezh2 *in 21 KB1P and 32 KP mammary tumors were obtained from oligonucleotide microarrays representing 18,173 genes. Methods for RNA extraction, RNA amplification, microarray hybridization, and data processing are described by Liu and colleagues [12]. For comparison of *EZH2 *gene expression (Log10 ratio) signatures between mouse and human breast tumors, we used the expression profiles of 96 human breast tumors: 18 ER-negative *BRCA1 *tumors, 34 tumors with a good prognosis signature and 44 tumors with a poor prognosis signature categorized by the human 70-genes signature dataset [31].

### Growth inhibition assays

DZNep was provided by YU Qiang (Genome Institute of Singapore) and trichostatin A (TSA) was obtained from Sigma-Aldrich (St. Louis, MO, USA). Both compounds were solved in DMSO and stored at -20°C. Before the growth inhibition assays, growth curves were made of all cell lines to determine the amount of cells that ensure exponential growth during a five-day culture period. For cell viability analysis, subconfluent dishes were trypsinized and filtered to obtain single cells. Cells were counted with a Casy counter and appropriate amounts of cells were typically plated out in 96-well plates on day 0. Drugs were added in twofold serial dilutions on day 1 in triplicate. DMSO was used as a no-drug control. Both drugs were left on the cells for 120 hours. On day 5 cell viability was measured using the Cell Titer Blue assay (Promega, Madison, WI, USA). Cell Titer Blue reagent was added directly to the cells (10 μl/well) and incubated for two hours at 37°C. Fluorescence at 560_ex_/590_em _nm was measured using a Tecan infinite m200 plate reader (Tecan, Salzburg, Austria). After correction for medium only and no-drug controls, data points were fitted to a sigmoidal dose-response curve with variable slope using GraphPad Prism Version 5.00 (GraphPad Software, San Diego, CA, USA): Y = 100/(1 + 10^((LogIC50-X)*HillSlope))). At least three independent experiments were used to determine the half maximal inhibitory concentration (IC_50_) values for each drug/cell line combination.

### RNA interference and DZNep-drug treatment

The SMARTpool small interfering RNA (siRNA) targeting *Ezh2 *and the non-targeting control were purchased from Dharmacon (Lafayette, CO, USA). Prior to all knock-down experiments optimal transfection conditions were determined for all cell lines. Cells were plated on day 0 and either transfected with 2 μM siRNA using DharmaFECT transfection reagent according to manufacturer's protocol (Dharmacon, Lafayette, CO, USA) or supplied with 5 μM DZNep on day 1. For protein and RNA analysis cells were harvested 48, 72 and 96 hours after transfection. The effect on cell growth was quantified using a Cell Titer Blue cell viability assay as described above. Cells were plated on day 0 in a density to allow exponential growth during the whole experiment and either transfected with siRNA or treated with 5 μM DZNep on day 1. Fluorescence was recorded 24, 48, 72 and 96 hours after transfection. Cell culture images were obtained using a Zeiss Axiovert 25 microscope with 10× objective on a Sony Cybershot. In all cases, data are presented from at least three independent experiments.

## Results

### Ezh2 expression is elevated in BRCA1-deficient mouse mammary tumors

**Figure 1:**
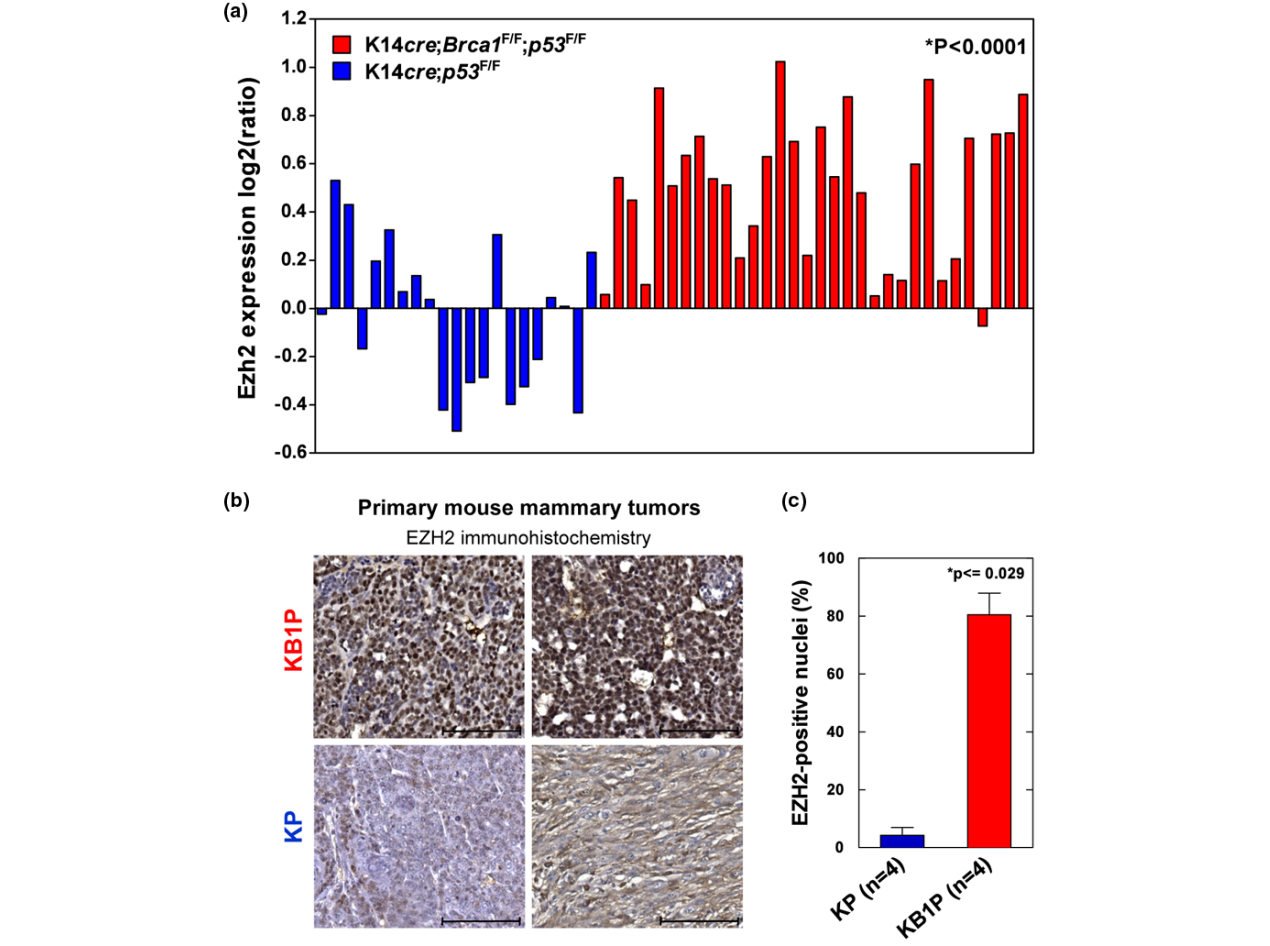
*Ezh2 *expression is elevated in BRCA1-deficient primary mouse mammary tumors. **(a) **mRNA levels of *Ezh2 *in BRCA1-deficient (*K14cre;Brca1*^*F*/*F*^*;p53*^*F*/*F *^(KB1P)) and BRCA1-proficient (*K14cre;Brca1*^*w*.*t*/*w*.*t*^*;p53*^*F*/*F *^(KP)) mammary tumors analyzed by microarray analysis. The mean (± standard error of the mean) log2 ratio of *Ezh2 *expression in 21 KP tumors is -0.036 (± 0.067) and 0.497 (± 0.054) in 32 KB1P tumors. The *Ezh2 *expression is significantly higher in KB1P tumors compared with KP tumors (*Wilcoxon exact test). **(b) **EZH2 protein levels in two independent primary KB1P and in two independent primary KP tumors detected by immunohistochemistry (scale bar represents 100 μm), representative of a total of four tumors analysed for each genotype. **(c) **Quantification of EZH2 immunohistochemistry shown in b (* Wilcoxon exact test).

To define the molecular changes associated with BRCA1-deficient breast cancer, we previously compared BRCA1-deficient (KB1P) mammary tumors derived from our conditional *K14cre;Brca1*^*F*/*F*^*;p53*^*F*/*F *^mouse model for hereditary breast cancer with BRCA1-proficient mammary tumors (KP) derived from *K14cre;Brca1*^*w*.*t*/*w*.*t*^*;p53*^*F*/*F *^mice. Gene expression microarray analysis showed that KB1P tumors expressed markers of basal-like breast cancer, for example p63 and keratin 5, compared with the KP tumors [12,32]. Strikingly, the polycomb repressor *EZH2 *is also higher expressed in BRCA1-deficient tumors than in BRCA1-proficient control tumors (Figure 1a). Whereas there is heterogeneity in the BRCA1-proficient group, virtually all BRCA1-deficient tumors display increased *Ezh2 *expression, suggesting that in the absence of BRCA1 increased levels of EZH2 may be required. To determine whether the increase in mRNA levels translates to higher EZH2 protein expression, we analyzed tissue sections from both KB1P and KP tumors by immunohistochemistry (Figure 1b). We indeed found that BRCA1-deficient mouse mammary tumors have higher EZH2 protein levels than control tumors, also indicated by the higher percentage of tumor cells with EZH2 expression above background (77% in KB1P tumors versus 11.5% in KP tumors; Wilcoxon *P *< 0.029; Figure 1c).

### EZH2 is overexpressed in BRCA1-deficient human breast tumors

**Figure 2:**
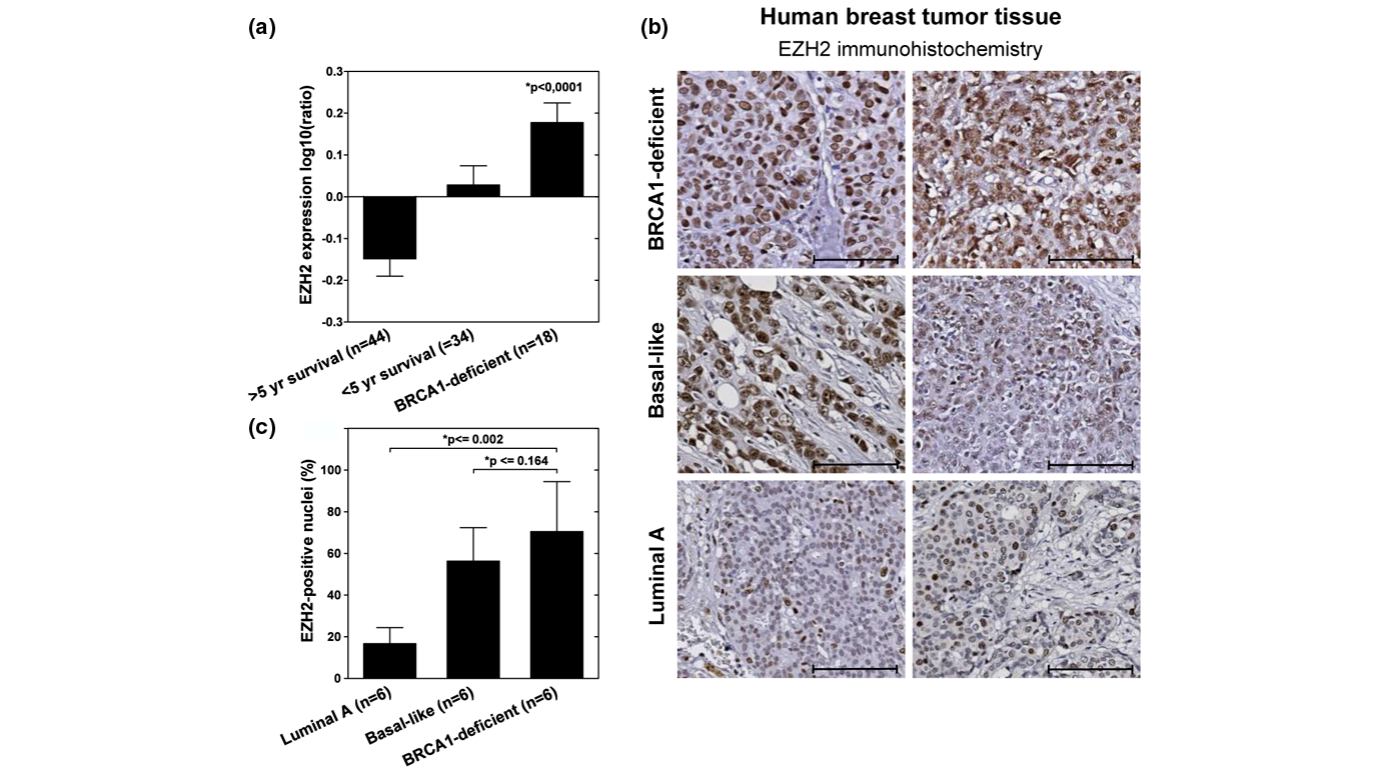
*EZH2 *is overexpressed in BRCA1-deficient human breast tumors. **(a) **The mean (± standard error of the mean) log10 ratio of *EZH2 *expression in human breast cancer samples [45] is -0.15 (± 0.041; >five-year survival, good prognosis), 0.028 (± 0.046; <five-year survival, poor prognosis) and 0.177 (± 0.047; BRCA1-deficient). *EZH2 *expression is significantly different between the three groups (* Kruskall-Wallis test). **(b) **Immunohistochemistry for EZH2 in human breast tumor tissues (scale bar represents 100 μm). Two examples are shown of individual tumors for each subtype, representative of a total of six tumors analysed per subtype. **(c) **Quantification of EZH2 immunohistochemistry shown in b (* Wilcoxon exact test).

Next, we determined whether EZH2 is also overexpressed in human BRCA1-deficient breast cancer. Recently, we and others showed that EZH2 levels are high in breast tumors with a poor prognosis [16,18,33]. Tumors from *BRCA1*-mutation carriers belong to this group of aggressive breast cancer, and accordingly *EZH*2 mRNA levels are also high in human BRCA1-deficient tumors (Figure 2a). Consistent with our previous observation that EZH2 mRNA and protein levels correlate relatively well [16], we observed increased EZH2 protein levels in human BRCA1-deficient tumor sections compared with other breast tumors (Figure 2b). To allow direct comparison, we simultaneously performed immunohistochemistry for EZH2 on sections from luminal A, basal-like and *BRCA1*-mutated breast tumors. As previously reported [16,18,33], we found EZH2 levels to be higher in basal-like tumors compared with luminal A-type tumors (Figure 2b). By the same detection criteria, EZH2 levels in the BRCA1-deficient tumors were at least as high as in the sporadic basal-like tumors (no significant difference, Wilcoxon *P *< 0.164) and significantly increased compared with luminal A-type tumors (Wilcoxon *P *< 0.002, Figure 2c).

### BRCA1-deficient cells are dependent on EZH2

To determine whether the increased EZH2 levels are functionally relevant in the BRCA1-deficient tumor cells, we made use of cell lines that were derived from KB1P and KP mouse mammary tumors [23]. In total, we used a panel of three BRCA1-deficient and three BRCA1-proficient cell lines, all derived from individual primary tumors. Although DZNep is a selective inhibitor of EZH2 function, some effects on other epigenetic marks, such as H4K20 methylation, have been reported [22]. To ascertain whether observed effects with DZNep are due to its inhibition of EZH2 specifically, we included siRNAs targeting *Ezh2 *in our experiments.

**Figure 3:**
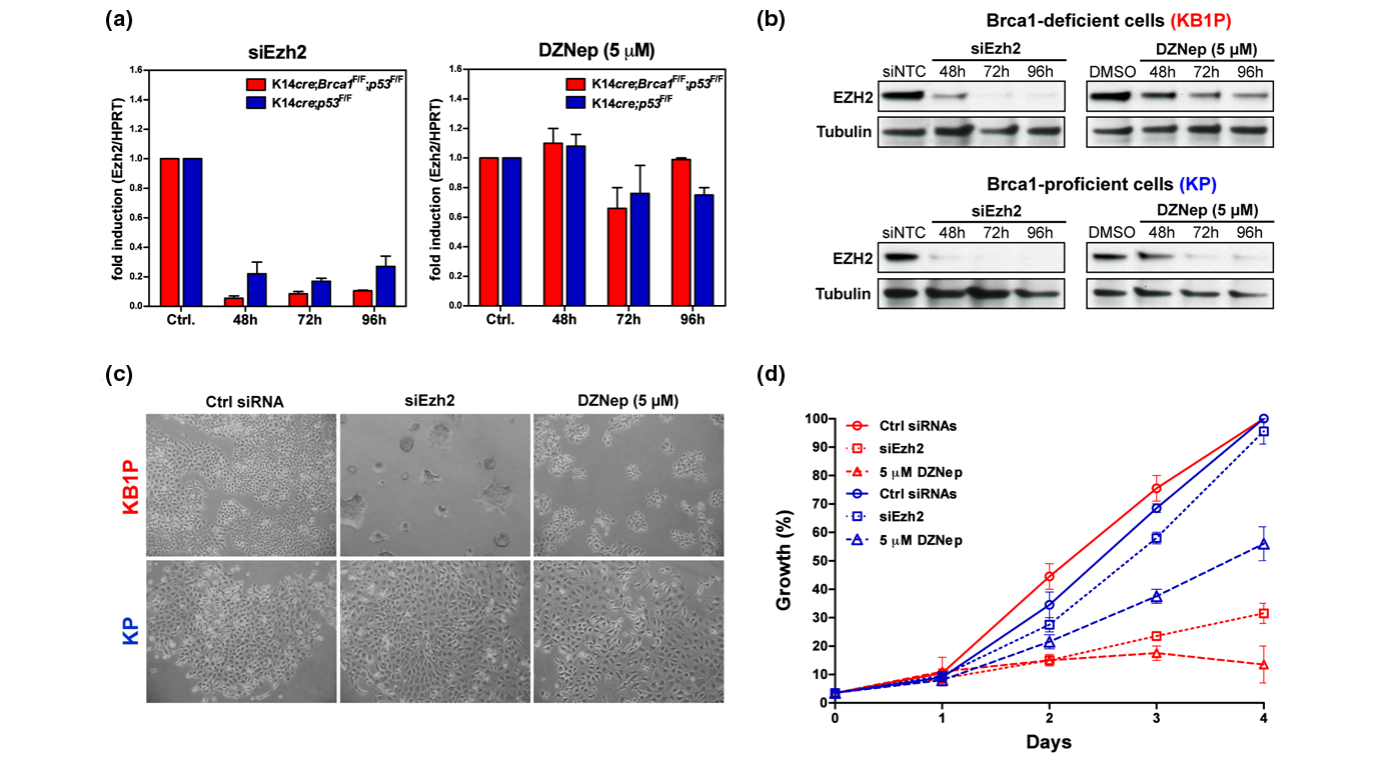
EZH2 is required for survival of BRCA1-deficient but not BRCA1-proficient tumors cells. **(a) ***Ezh2 *mRNA expression measured by quantitative RT-PCR in BRCA1-deficient cells (in red, *K14cre;Brca1*^*F*/*F*^*;p53*^*F*/*F *^(KB1P)) and in BRCA1-proficient cells (in blue, *K14cre;Brca1*^*w*.*t*/*w*.*t*^*;p53*^*F*/*F *^(KP)) after treatment with siRNAs against *Ezh2 *or 5 μM 3-deazaneplanocin A (DZNep; shown as fold induction relative to *Hprt*). **(b) **EZH2 levels analysed by western blot after KB1P and KP cells were treated with siRNAs targeting *Ezh2 *or non-targeting siRNAs (siNTC) and 5 μM DZNep or vehicle (dimethyl sulfoxide (DMSO)) for the indicated period. **(c) **Phase-contrast images of BRCA1-deficient and BRCA1-proficient cells treated for 48 hours with control (ctrl) siRNAs, siRNAs against *Ezh2 *or 5 μM DZNep (original magnification 10×). **(d) **Growth curves of KB1P (depicted in red) and KP cell lines (depicted in blue) treated with control (ctrl) siRNAs, siRNAs against *Ezh2 *or 5 μM DZNep. Data measured by Cell Titer Blue and represented as the mean ± standard error of the mean (three independent experiments). The depicted cell lines are representative for all three KB1P and KP cell lines.

Quantitative PCR demonstrated efficient knockdown of *Ezh2 *mRNA levels in both BRCA1-proficient and BRCA1-deficient cells (Figure 3a). DZNep does not affect *Ezh2 *mRNA levels, as reported previously [22]. However, treatment with either *Ezh2*-specific siRNAs or DZNep resulted in significant reduction of EZH2 protein levels in both KB1P and KP cells (Figure 3b). Forty-eight hours after treatment there was visible toxicity in KB1P cells when EZH2 levels were reduced by either DZNep or siRNAs targeting *Ezh2*, but not in KB1P cells treated with non-targeting control siRNAs (Figure 3c). In contrast, there was no apparent effect of reduced EZH2 levels, either by *Ezh2 *knock-down or DZNep treatment, in BRCA1-proficient tumor cells. A more quantitative assessment of the effect of the treatments by a growth inhibition assay revealed that there is some adverse affect of DZNep in the KP cells. However, this effect of DZNep is unrelated to EZH2, as knock-down of *Ezh2 *does not inhibit the growth of these cells (Figure 3d, blue lines). Possibly, this is due to the effect of DZNep on H4K20 or other methylation events. In contrast, KB1P cells are severely affected by reduced EZH2 levels, as demonstrated by a strong growth inhibition of KB1P cells treated with siRNAs targeting *Ezh2 *(Figure 3d, red lines). In BRCA1-deficient cells, treatment with DZNep inhibited growth even more effectively than knock-down of *Ezh2*, which could be due to a more effective depletion of EZH2 by DZNep than that achieved by siRNAs, or due to possible effects of DZNep on other epigenetic marks. Nevertheless, DZNep shows remarkable selectivity in inhibiting BRCA1-deficient tumor cells compared with BRCA1-proficient tumor cells (Figures 3c, d).

### BRCA1-deficiency sensitizes cells to EZH2-inhibitor DZNep but not to TSA

**Figure 4:**
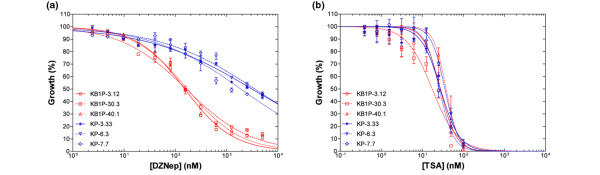
Chemical EZH2-inhibitor DZNep selectively kills BRCA1-deficient tumor cells. **(a) **Representative growth inhibition curves for BRCA1-deficient cell lines (*K14cre;Brca1*^*F*/*F*^*;p53*^*F*/*F *^(KB1P), in blue) and BRCA1-proficient cell lines (*K14cre;Brca1*^*w*.*t*/*w*.*t*^*;p53*^*F*/*F *^(KP), in red) treated with 3-deazaneplanocin A (DZNep). A serial dilution of DZNep was added to the cells and cell viability was measured five days later (each data point represents the mean ± standard error of the mean (SEM) of three independent experiments). **(b) **Representative growth inhibition curves for BRCA1-deficient cell lines (KB1P, in blue) and BRCA1-proficient cell lines (KP, in red) treated with trichostatin A (TSA). A serial dilution of TSA was added to the cells and cell viability was measured five days later (mean ± SEM).

To better quantify the difference in sensitivity to DZNep between KB1P and KP cells, we performed a dose-response curve (Figure 4a). Strikingly, the average IC_50 _for BRCA1-deficient cells is 163 nM, whereas an almost 19-fold higher dose is required for 50% growth inhibition in BRCA1-proficient cells (average IC_50 _of 2944 nM, *P *< 0.0001, t-test; Table 1).

**Table 1:** IC_50 _values of DZNep and TSA on BRCA1-proficient vs. BRCA1-deficient mammary tumor cells

**Cell line**	**nM DZNep (SEM)**	**nM TSA (SEM)**
***BRCA1-proficient***		
KP-3.33	3202 (284)	27 (1.2)
KP-6.3	3467 (277)	35 (1.5)
KP-7.7	2163 (63)	25 (3.8)
***BRCA1-deficient***		
KB1P-3.12	154 (14)	26 (2.4)
KB1P-30.3	163 (13)	17 (0.6)
KB1P-40.1	171 (9)	36 (3.4)

**Ratio (KP/KB1P)**	18.1*	1.1
**Significance**	< 0.0001	0.5

Note: Values between brackets represent the standard error of the mean (SEM) of at least three independent experiments. Ratio: Average half maximal inhibitory concentration (IC_50_) values of 3-deazaneplanocin A (DZNep) and trichostatin A (TSA) for the *K14cre;Brca1*^*w*.*t*/*w*.*t*^*;p53*^*F*/*F *^(KP) lines were compared with average IC_50 _values of the *K14cre;Brca1*^*F*/*F*^*;p53*^*F*/*F *^(KB1P) lines. *Significance: *P *< 0.01 (t-test).

To exclude the possibility that KB1P cells are in general more sensitive to epigenetic inhibitors we tested the effect of the histone deacetylase-inhibitor TSA in the same growth inhibition assay (Figure 4b). TSA affected KB1P and KP cell lines to a similar extend (average IC_50 _of 26 nM vs 29 nM) showing no significant difference (*P *= 0.5; t-test, Table 1). When the cell lines were grown under non-adherent conditions, DZNep also inhibited sphere-formation, suggesting that there is no subpopulation of BRCA1-deficient cells that is resistant to DZNep treatment [see Additional data file 1]. However, *in vivo *experiments should demonstrate whether targeting EZH2 inhibits all tumor-initiating potential.

### Reconstitution of BRCA1 partially restores resistance to DZNep

**Figure 5:**
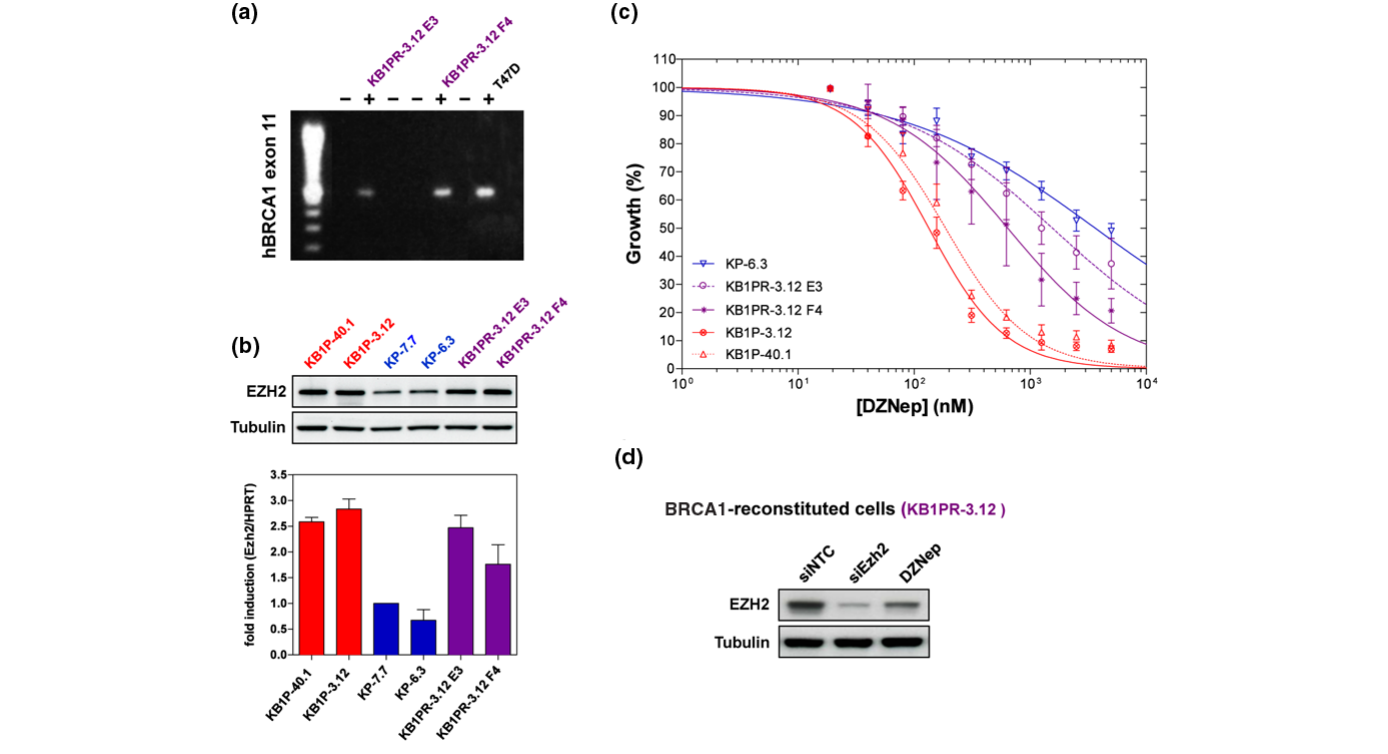
Restoration of BRCA1 partially rescues from sensitivity to DZNep. **(a) **Detection of the human *BRCA1 *allele by PCR amplification of exon 11 in the reconstituted subclones KB1PR-3.12 E3 and F4. The human breast cancer cell line T47D was used as a positive control. **(b) **EZH2 protein levels of (untreated) *KK14cre;Brca1*^*F*/*F*^*;p53*^*F*/*F *^(KB1P), KB1PR and *K14cre;Brca1*^*w*.*t*/*w*.*t*^*;p53*^*F*/*F *^(KP) cell lines were analysed by western blotting. Corresponding *Ezh2 *mRNA levels of KB1P, KB1PR and KP cell lines were measured by quantitative RT-PCR (shown as fold induction relative to *Hprt*). **(c) **Representative growth inhibition curves for BRCA1-deficient cell lines (KB1P, in blue), BRCA1-proficient cell lines (KP, in red) and two clones of a *BRCA1*-reconstituted cell line (KB1PR E3 and F4, in purple) treated with 3-deazaneplanocin A (DZNep). A serial dilution of DZNep was added to the cells and cell viability was measured five days later (mean ± standard error of the mean). **(d) **EZH2 protein levels of *hBRCA1*-reconstituted KB1P cells 48 hours after treatment with DZNep or siRNAs targeting EZH2.

As loss of BRCA1 function results in genomic instability, we wanted to establish whether the dependence on EZH2 is a direct consequence of *Brca1 *loss, or whether this is a secondary effect caused by mutations accumulated during the tumorigenic process. To test this, we re-introduced a BAC clone encompassing the complete human *BRCA1 *gene into a BRCA1-deficient cell line (KB1P-3.12) and derived several clones that were shown to re-express *BRCA1 *(Figure 5a). These cells became less sensitive to cisplatin treatment indicating that the introduced BRCA1 is functional (data not shown). Of note, we did not observe a decrease in EZH2 levels in the reconstituted cell lines, indicating that BRCA1 does not directly influence *Ezh2 *expression (Figure 5b). However, treatment with DZNep reduces EZH2 levels to a similar extent in all cell lines (Figures 3b and 5d, and data not shown).

Interestingly, when the reconstituted cell lines were treated with DZNep, we observed a substantial rescue from DZNep-induced cell death (Figure 5c). The IC_50 _values for DZNep in the *BRCA1*-reconstituted cells lines were more similar to the IC_50 _values of the KP cells, than those of KB1P cells (average IC_50 _of 1391 nM, Table 2). This shows that sensitivity of BRCA1-deficient cells to DZNep is mainly due to a loss of BRCA1 function, and not due to secondary mutations. This also indicates that there is a synthetic lethal effect; the effect of targeting one gene (e.g. *EZH2*) becomes deleterious specifically in the absence of another gene (e.g. *BRCA1*) [34].

**Table 2:** IC_50 _values of DZNep on BRCA1-deficient vs. BRCA1-reconstituted mammary tumor cells

**Cell line**	**nM DZNep (SEM)**
***BRCA1-proficient***	
KP-6.3	3523 (463)
***BRCA1-reconstituted ***	
KB1PR-3.12 E3	1954 (744)
KB1PR-3.12 F4	829 (331)
***BRCA1-deficient***	
KB1P-3.12	135 (13)
KB1P40.1	187 (33)

**Ratio (BRCA1-reconst. vs. deficient)**	8.7*
**Significance**	0.003

Note: Values between brackets represent the standard error of the mean (SEM) of at least three independent experiments. Ratio: Average half maximal inhibitory concentration (IC_50_) values of 3-deazaneplanocin A (DZNep) for the *K14cre;Brca1*^*F*/*F*^*;p53*^*F*/*F *^(KB1P) lines were compared with average IC_50 _values of the KB1P lines. *Significance: *P *< 0.01 (t-test).

Although the reconstituted cells become over eight-times more resistant to DZNep, the rescue is not complete. This could be due to differences in mouse and human *BRCA1 *or to technical issues with achieving the correct amount of *BRCA1 *expression. Alternatively, additional mutations could play a minor role in the sensitivity to DZNep.

In summary, we have demonstrated that DZNep selectively inhibits BRCA1-deficient but not BRCA1-proficient mammary tumor cells, and that this effect is mainly due to the fact that BRCA1-deficient cells are dependent on EZH2, whereas BRCA1-proficient cells are not.

## Discussion

Breast tumors in *BRCA1*-mutation carriers arise early in life and exhibit an aggressive, basal-like phenotype associated with poor overall survival. More insight into the molecular make-up of this breast cancer subtype will contribute to the development of more effective therapies. In this study, we demonstrate that EZH2 expression is high in breast tumors from *BRCA1*-mutation carriers, similar to that observed in our mouse model for BRCA1-deficient breast cancer. Moreover, the knock-down experiments show that BRCA1-deficient mammary tumor cells are dependent on EZH2 for their survival. Interestingly, although EZH2 levels were also reduced to a similar level in the BRCA1-proficient control cells, these cells seem much less affected by EZH2 loss. This indicates that targeting EZH2 is synthetic lethal in combination with BRCA1-deficiency. Conceivably, the dependence on high EZH2 levels derives from a selective advantage during the *in vivo *tumorigenesis process that occurs only in BRCA1-deficient and not BRCA1-proficient cells. The observation that restoration of BRCA1 does not reduce EZH2 levels suggests that the increased expression is caused by more permanent changes. However, such mutations or epigenetic alterations do not necessarily have to target *Ezh2 *directly, but could occur in upstream regulators of EZH2 as well. Note that selection for *Ezh2 *overexpression may occur in other breast tumors, as evident from some of the primary BRCA1-proficient mouse tumors in Figure 1a. The central question of this study was whether overexpression of EZH2 is required for the survival of breast tumor cells or whether this is a byproduct of the tumorigenic process, and our data suggest that whereas BRCA1-deficient cells remain dependent on their EZH2 expression, loss of EZH2 is much better tolerated in cells with intact BRCA1.

Now that we have established the functional importance of EZH2 expression in BRCA1-deficient cells, it would be interesting to understand why these tumors are selectively dependent on EZH2, and whether this dependence is specific to loss of BRCA1 function, or more related to the basal-like characteristics of these tumors. There could be several, not mutually exclusive, reasons for selection of *EZH2/Ezh2 *overexpression during tumorigenesis.

With regards to a specific role of BRCA1-deficiency, there could be an effect of EZH2 on DNA repair. It has been reported that *EZH2 *overexpression represses genes of the Rad51 family [35], which may attenuate DNA damage signalling in BRCA1-deficient cells. This would suggest that *EZH2 *overexpression could play a similar role in BRCA2-deficient tumors that are subject to the same impairment in homology-directed double-strand break repair as BRCA1-deficient tumors. However, we did not observe increased EZH2 expression in breast tumors from *BRCA2*-mutation carriers (data not shown), suggesting that the main oncogenic role of EZH2 is not linked to DNA repair. Another possible explanation for the selective overexpression of EZH2 in BRCA1-deficient breast tumors could involve a role of EZH2 in the cell of origin. Specifically, the absence of BRCA1 has been associated with characteristics of stem cells and loss of BRCA1 is incompatible with luminal differentiation [11]. EZH2 is required for the maintenance of embryonic and adult stem cells, is expressed in a relatively small number of cells in the mammary gland, and is only overexpressed in breast tumors with an undifferentiated phenotype [16,19,36]. In addition, a large subset of genes silenced by EZH2 includes transcription factors that orchestrate lineage-specific differentiation [37,38]. Therefore, it could be envisaged that overexpression of *EZH2 *is required to maintain the undifferentiated state of the transformed cell. Reducing EZH2 levels by DZNep or siRNAs might result in the expression of genes that induce differentiation, a fate potentially incompatible with the absence of BRCA1.

However, overexpression of *EZH2 *does not seem to cause hyperrepression of typical PcG-target genes [16,39], suggesting that it has consequences distinct from silencing its normal target genes. Several groups have indeed found evidence for genes marked by PcG proteins specifically in tumor cells [22,40]. It remains to be established, however, whether silencing of these genes is responsible for the selective advantage of *EZH2 *overexpression in BRCA1-deficient tumor cells, and whether these genes include more classical tumor-suppressors or specific differentiation factors.

The model for a specific function of EZH2 in more undifferentiated, basal-like cells is consistent with the observation that our KB1P tumors are indeed more basal than the KP control tumors [12]. This raises the question if not only BRCA1-deficient breast tumors but also sporadic basal-like tumors would be dependent on *EZH2 *overexpression. Our observation that restoration of BRCA1 function negates the sensitivity of tumor cells to DZNep would argue against this. However, although *BRCA1 *mutations in sporadic cancer are rare, there are indications that a significant proportion of sporadic breast tumors share traits with BRCA1-deficient tumors, a feature termed BRCAness [41]. Alterations in genes functioning in the same biochemical pathways as BRCA1 could effectively result in loss of BRCA1 function [9]. Sporadic basal-like tumors that exhibit this feature may be specifically sensitive to EZH2 inhibition. Recently, Gonzalez and colleagues [42] showed that knock-down of *EZH2 *reduced the proliferation of two ER-negative human breast cancer cell lines. Intriguingly, this effect seemed partly due to an upregulation of BRCA1 protein levels. This is in disagreement with our data which show that cells without BRCA1 are particularly sensitive to EZH2 reduction, suggesting that repression of *Brca1 *is not the main oncogenic function of EZH2. Moreover, breast tumors in *BRCA1*-mutation carriers show invariably loss of the other *BRCA1 *allele, indicating that selection for loss of *BRCA1 *expression is not achieved by high levels of EZH2. However, the observation that these two basal-like breast cancer cell lines are also sensitive to EZH2 inhibition, and the repeated observation that *EZH2 *overexpression characterizes basal-like breast tumors, warrants the further investigation of EZH2 as a druggable target.

Unfortunately, our preliminary *in vivo *studies with DZNep revealed substantial toxicity in mice (data not shown). We are currently investigating whether this is due to a dependence of certain normal cell types on *EZH2 *expression, or whether this is due to chemical properties of DZNep. The former would complicate EZH2 inhibition as therapeutic strategy in breast cancer, whereas the latter may be resolved by using other EZH2 inhibitors or DZNep-analogs, which are currently being developed. In addition, although DZNep inhibits sphere-formation of BRCA1-deficient tumor cells and considering the role of EZH2 in stem cells and cancer [43], *in vivo *studies will be required to determine whether targeting of EZH2 by itself or in combination with other treatments can result in complete eradication of the tumor.

## Conclusions

The preclinical studies and clinical trials with combinations of platinum drugs and Poly(ADP-ribose) polymerase (PARP) inhibitors against *BRCA1*-mutated breast cancers constitute one example of how insight into the genetic make-up of a tumor subtype can provide a targeted and possibly more effective treatment [34,44]. Our data show that BRCA1-deficient tumor cells are selectively dependent on EZH2 expression, and suggest that pharmacological disruption of EZH2 could provide another individualized approach for the treatment of *BRCA1*-mutated breast cancers, and possibly also for sporadic basal-like breast tumors.

## Abbreviations

BAC: bacterial artificial chromosome; BRCA1: breast cancer 1 gene; BSA: bovine serum albumin; DAB: diaminobenzidine; DMEM: Dulbecco's Modified Eagle Medium; DMSO: dimethyl sulfoxide; DZNep: 3-deazaneplanocin A; ER: estrogen receptor; EZH2: human homolog of the Drosophila enhancer of zeste gene; FCS: fetal calf serum; HER: human epidermal growth factor receptor; HRP: horseradish peroxidase; IC50: half maximal inhibitory concentration; KB1P: *K14cre;Brca1*^*F*/*F*^*;p53*^*F*/*F*^, cells derived from mammary tumors arising in mice with this genotype are BRCA1-deficient; KP: *K14cre;Brca1*^*w*.*t*/*w*.*t*^*;p53*^*F*/*F*^, cells derived from mammary tumors arising in mice with this genotype are BRCA1-proficient; PARP: Poly(ADP-ribose) polymerase; PBS: phosphate-buffered saline; PcG: Polycomb Group; PR: progesterone receptor; PRC2: Polycomb Repressive Complex 2; RT-PCR: reverse-transcription polymerase chain reaction; SCM: stem cell medium; siRNA: small interfering RNA; TSA: trichostatin A.

## Competing interests

The authors declare that they have no competing interests.

## Authors' contributions

AP initiated the study, conceived the design with MvL and JJ, and wrote the manuscript with JP. JP participated in the design and performed most of the experiments and data analysis. RD performed the experiments with the reconstituted cell lines she created. XL provided the panel of cell lines and micro-array data. BE advised on the set-up and analysis of the IC_50 _studies. PC performed western blots and quantitative PCRs. PN provided human tumor samples. SA participated in the immunohistochemical analysis of those samples. QY provided the DZNep. MvL and JJ gave expert advice throughout the study. All authors read and approved the final manuscript.

## Supplementary Material

Additional file 1 A PDF file containing a figure that shows that DZNep prevents sphere-formation in three independent BRCA1-deficient cell lines. Phase contrast images are shown of KB1P-3.12, 30.3 and 40.1 cells three days after plating in serum-free medium with defined growth factors on ultra-low binding plates in the presence of 5 μM 3-deazaneplanocin A (DZNep) or dimethyl sulfoxide (DMSO). Click here for file
