# Supplementary figures and images for: BRCA1-deficient mammary tumor cells are dependent on EZH2 expression and sensitive to Polycomb Repressive Complex 2-inhibitor 3-deazaneplanocin A
Source: Breast Cancer Res. 2009 Aug 26;11(4):R63. doi: 10.1186/bcr2354 (PMC2750125; doi:10.1186/bcr2354)

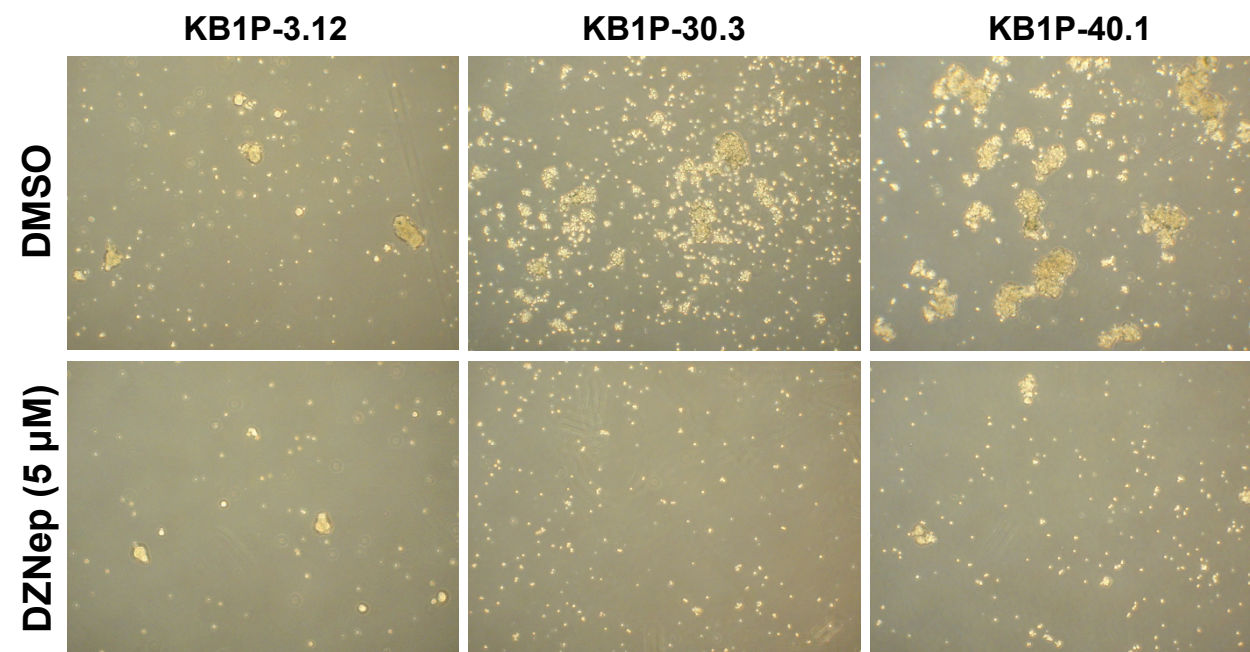

Supplement: Additional file 1 — A PDF file containing a figure that shows that DZNep prevents sphere-formation in three independent BRCA1-deficient cell lines. Phase contrast images are shown of KB1P-3.12, 30.3 and 40.1 cells three days after plating in serum-free medium with defined growth factors on ultra-low binding plates in the presence of 5 μM 3-deazaneplanocin A (DZNep) or dimethyl sulfoxide (DMSO). [file bcr2354-S1.pdf]
